# The financing need for expanded maternity protection in Indonesia

**DOI:** 10.1186/s13006-019-0221-1

**Published:** 2019-06-25

**Authors:** Adiatma Y. M. Siregar, Pipit Pitriyan, Dylan Walters, Matthew Brown, Linh T. H. Phan, Roger Mathisen

**Affiliations:** 1 Center for Economics and Development Studies, Department of Economics, Faculty of Economics and Business, Universitas Padjadjaran, Jl. Hayam Wuruk 6–8, Bandung, West Java 40115 Indonesia; 2 Canadian Centre for Health Economics, Institute of Health Policy, Management and Evaluation, University of Toronto, and Nutrition International, Ottawa, Ontario Canada; 3 Alive & Thrive, Southeast Asia, 7F, Opera Business Center, 60 Ly Thai To Street, Hanoi, Vietnam

**Keywords:** Breastfeeding, Maternity protection, Maternity leave, Inadequate breastfeeding, Economic analysis, Indonesia

## Abstract

**Background:**

Almost half of all Indonesian children under 6 months of age were not exclusive breastfed in 2017. Optimizing maternity protection programs may result in increased breastfeeding rates. This study aims to: estimate the potential cost implications of optimizing the current paid maternity protection program, estimate budgets needed to increase coverage of lactation rooms in mid and large firms, and explore challenges in its implementation in Indonesia.

**Methods:**

The potential cost implication of the current and increased maternity leave length (three and 6 months) as well as the potential budget impact to the government were estimated for 2020 to 2030. The cost of setting up lactation rooms in formal sector companies was estimated using the Alive & Thrive standards. Interviews were conducted in five different provinces to 29 respondents in 2016 to identify current and potential challenges in implementing both existing and improved maternity protection policies.

**Results:**

The costs of expanding paid maternity leave from three to 6 months and incorporating standardized lactation rooms in 80% of medium and large size firms in Indonesia was estimated at US$1.0 billion (US$616.4/mother per year) from 2020 to 2030, covering roughly 1.7 million females. The cost of setting up a basic lactation room in 80% of medium and large companies may reach US$18.1 million over 10 years. The three main barriers to increasing breastfeeding rates were: breastmilk substitutes marketing practices, the lack of lactation rooms in workplaces, and local customs that may hamper breastfeeding according to recommendations.

**Conclusions:**

The cost of expanding paid maternity leave is lower than the potential cost savings of US$ 1.5 billion from decreased child mortality and morbidity, maternal cancer rates and cognitive loss. Sharing the cost of paid maternity leave between government and the private sector may provide a feasible economic solution. The main barriers to increasing breastfeeding need to be overcome to reap the benefits of recommended breastfeeding practices.

**Electronic supplementary material:**

The online version of this article (10.1186/s13006-019-0221-1) contains supplementary material, which is available to authorized users.

## Background

Policies that ensure maternity protection programs that include paid maternity leave, are important to safeguard the health and livelihood of women and children [1]. Recent evidence suggests that maternity protection is associated with higher rates of breastfeeding and vaccinations in low and middle income countries [2, 3]. Longer paid maternity leave may reduce infant deaths [4, 5]. With more women entering the workforce [6], governments need to adapt policies to ensure that employed mothers and families are able to provide essential care during the 1000 days’ period of life, which includes pregnancy and infancy stages of life, without sacrificing income and employment opportunities.

In 2017, 52% of Indonesian infants under 6 months received exclusive breastfeeding as recommended by the World Health Organization (WHO)/United Nation’s Children Fund (UNICEF) guidelines [7]. Exclusive breastfeeding under 6 months is defined as the proportion of infants 0–5 months of age who are fed exclusively with breast milk, calculated as the number of infants 0–5 months of age who received only breast milk during the previous day divided by infants 0–5 months of age [8]. This means that almost half of all Indonesia children receives inadequate breastfeeding and nutrition care in the first months of life. Increased breastfeeding in Indonesia could prevent approximately 5377 child deaths each year, which is roughly equal to the total number of deaths by similar cause in Cambodia, Laos, Myanmar, Thailand, Timor-Leste, and Viet Nam combined [9].

The Government of Indonesia has implemented various regulations in support of maternity protection. The Ministry of Labor and Transmigration has issued a law that requires both public and private companies to provide 13 weeks of maternity leave and pay 100% salary during the leave period [10]. The Government Regulation no. 33/2012 states that companies should support exclusive breastfeeding and provide a lactation space, and further declares that companies not applying this regulation will be penalized [11]. The latter regulation also states that funding required to support exclusive breastfeeding can come from the national, regional, and other sources of budget [11]. Within the Government of Indonesia, the Ministry of Women Empowerment and Child Protection is responsible for increasing the knowledge of female workers and all employers about breastfeeding, and the Ministry of Labor and Transmigration is responsible for pressing companies and unions to support, regulate and promote breastfeeding in the workplace. The Ministry of Health is responsible for providing training on breastfeeding to workers as well as information and education to increase breastfeeding [12]. Several regulations have also been instituted to curtail the marketing of breast milk substitutes (BMS) in order to protect exclusive and continued breastfeeding for 2 years [11, 13–17], and Aceh province has put in place a governor regulation to extend maternity leave to 6 months for government employees [18].

However, there are still limitations to the policy regarding maternity protection in Indonesia. The existing policy on maternity leave provides 100% of salary for 13 weeks of paid maternity leave [10], and not the minimum 18 weeks recommended by the International Labour Organization (ILO) [19], or the ideal period of 6 months to align with the recommended period for exclusive breastfeeding set by the WHO and UNICEF [20]. Based on an ILO report [1], these policies are not yet optimally enforced and currently only 4.5% of women in Indonesia who are eligible to receive paid maternity leave in the formal sector actually receive it. There is no formal assurance that those taking maternity leave will be able to return to their job after maternity leave [1]. Unclear company policies on using breaks for expressing breastmilk, limited access to workplace lactation rooms [1, 21, 22], and aggressive breastmilk substitutes (BMS) marketing targeting caregivers with young children all contribute to a lack of clarity and inhibit breastfeeding friendly workplace environments [11, 13–16]. Even if policies supporting breastfeeding exist, and firms do own friendly childcare, the policies’ and facilities’ role in supporting breastfeeding may not always be clearly communicated to the employees, requiring proper action and support from the government and all stakeholders involved to address the issue [23]. Indeed, even though Indonesia has developed relevant policies to support breastfeeding, the monitoring and implementation are still deemed ineffective [24].

The previous issues further complicate the nature of female employment and providing breastfeeding. Two recent studies in Indonesia show a trend toward lower rates of exclusive breastfeeding among working women [25, 26]. More specifically, the studies document that 29 - 38% of working women exclusively breastfeed per recommendations, compared to 42 to 44% of non-working mothers. Differentiating between formal and informal workers, 24 to 30% of women working in the formal sector exclusively breastfeed compared to 40 to 47% for those who work informally. Although the rates are relatively low for all working women, mothers working in the formal sector fare worse. Notwithstanding the higher rates of exclusive breastfeeding among mothers working in the informal sector compared to the formal sector, more than half of women in Indonesia who are working informally (58%) [27] do not breastfeed exclusively. Different policies are needed to protect these women since they are currently not covered by the regulations.

Action to address some of these issues through maternity leave and protection can increase breastfeeding and vaccination rates, which in turn promotes health, prevents diseases for children, and has the potential to save money (e.g. costs for parents, insurance companies, employers, and society in general) both in the short and long run [2, 3, 9, 28, 29]. Globally, national maternity protection programs adopt different levels of publicly financed maternity protection, cash benefits rates, and access to workplace lactation rooms [30]. As part of an investment framework for nutrition, the World Bank estimated that the cost to scale up the extension of maternity leave cash benefits to new mothers employed in the formal sector across low and middle income countries (LMICs) would cost at least US$24.1 billion over 10 years and avert 520,000 child deaths [31].

There is an urgent need to convince policymakers in Indonesia to provide more support for enforcing or creating stronger maternity protection programs. Evidence from economic research may help to inform policymakers on the costs and consequences of not investing in breastfeeding promotion and maternity protection. However, currently, little information is available that addresses the cost of maternity protection and the budget implication of creating lactation rooms in Indonesia.

This study aims to estimate the potential cost implications of expanding the paid maternity leave policy for all working women and of providing lactation rooms in workplaces in Indonesia through economic modelling using national and provincial data. In addition, results from in depth interviews that gathered qualitative data from key stakeholders are presented to support the quantitative result. All in all, this study seeks to address the four following research questions: first, what would it cost if 21% of eligible women actually received the leave to which they are entitled (as opposed to only 4.5%, currently)? Second, what would it cost to align the length of paid maternity leave from the current 13 weeks to the recommended level of 6 months for exclusive breastfeeding, and what would the cost be to the government to take on a majority share of this cost? Third, what is the cost of creating lactation rooms in workplaces across Indonesia? Fourth, what are the challenges experienced in implementing the existing breastfeeding and maternity policies?

It is important for policymakers to have access to accurate data on the cost of public sector programs, including maternity leave benefits, in order to adequately assess the potential budget impact as well as the cost-benefit of investing in the programs. It is also important to demystify political and public perceptions that social welfare and public health programs are prohibitively expensive. While this information may be most useful for policy-makers in Indonesia, the general findings may be applicable to other emerging countries and may stimulate interest in studying the topic in Southeast Asia and beyond.

## Methods

This section outlines the methodology used to estimate the cost of paid maternity leave and the creation of lactation rooms in workplaces, as well as to conduct stakeholder interviews on the barriers to implementation of maternity protection policies in Indonesia. The timeframe of this analysis is from 2020 to 2030, allowing for time for policy change to take place. This study was approved by the ethical committee of the Faculty of Medicine at the corresponding authors’ institute.

### Basic assumptions

Population growth data from the World Development Indicators (WDI) [32] was used to estimate the population between 2020 and 2030. Provincial-level population data from the 2010 Indonesian Census was used to estimate the population of children between 0 and 11 months of age [33] as a proxy for women who could be exclusively breastfeeding their infants and was adjusted to the year 2020 using the population growth data. The labor force participation rate (female ratio) from the WDI [6], the percentage of potential coverage of women who are eligible to receive paid maternity leave [1] and the percentage of women working in the formal sector [27] were used to estimate the potential number of women who will receive paid maternity leave in each province in year 2020 (amounting to 48,664 females, Additional file 1). The female labor force participation rate is assumed to increase by 0.20% per year up to 53.30% on the basis of the increase from the year 2016 to 2017 in WDI data. From the year 2020 to 2030 the wage per province is assumed to be constant to calculate real costs. These assumptions are presented in Table 1. All calculations were done in constant 2016 US$.

**Table 1:** Base and scenario assumptions

Items	Value used in base scenario	Value used in scenario 1 and 2		Values varied for sensitivity analysis
Period of estimation	2020–2030	2020–2030		–
Exchange rate (2016)	Rp 13,120/US$ [34]	Rp 13,120/US$ [34]		±25%
Rate of cash benefit, % of average wage, US$110.6^f^ [27]	100 [1]	100 [1]		–
Percentage of women working in formal sector [27]	42.12	42.12		–
Female labor participation rate (%)	51.30 [6]	51.30 [6]	2020	–
		up to^e^		
		53.30	2030	–
Potential coverage of women who are eligible to receive paid maternity leave (%)	4.5^a^	4.5^a^	2020	–
		up to^e^		
		21^b^	2030	9^c^ – 32^d^
Number of large and medium companies		24,529 [27]		
Percentage of large and medium companies with lactation rooms	n/a	10.5 [35]	2020	–
		up to^e^		
		80	2030	–

This table shows all of the base and scenario assumptions used in the calculation

Assumptions derived from ILO report [1]: ^a^Median of coverage in practice of maternity leave cash benefits, ^b^Median of coverage in law of maternity leave, ^c^Maximum coverage in practice of maternity leave cash benefits, ^d^Maximum of coverage in law of maternity leave, ^e^“Up to” refers to gradual yearly increase: see Table 2, ^f^this is national level average wage, only serves to give a rough picture of the amount for the readers. The wage rate used for the calculation itself was the average wage rate per province

### The financing need of paid maternity leave and collectively financed maternity benefits

The base scenario was the current cost of paid maternal leave using baseline assumptions, under which not all women receive the paid leave to which they are legally entitled. The financing need was calculated by multiplying the number of women covered (Table 2) with the average wage for each province, and with the length of the maternity leave (in base scenario, the length is 3 months). In scenario 1, the percentage of potential coverage of eligible women was increased from 4.5% in 2020 to 21% in 2030 on the basis of an ILO report [1]. In this scenario, the cost of paid maternity leave was estimated for 3 months of maternity leave. Scenario 2 replicates scenario 1, but the cost is estimated for 6 months of leave. We subsequently multiply this amount by two thirds (preferred share of cash benefit payment between government and employer) [1] to estimate potential costs to be borne by the government if they are to absorb a share, representing a collectively financed maternity leave benefit by the government and private companies. However, we do not estimate the budget regularly spent by the government to cover the maternity cash benefit of their employees in this study.

**Table 2:** Estimates of women in the formal sector receiving paid maternity leave as coverage is increased from 4.5 to 32% between 2020 and 2030 as adjusted for increased labor participation of women and population growth in Indonesia

Year	2020	2021	2022	2023	2024	2025	2026	2027	2028	2029	2030	Total
Female labor participation rate (%)	51.30	51.50	51.70	51.90	52.10	52.30	52.50	52.70	52.90	53.10	53.30	
Estimated number of women working in formal sector eligible to paid maternity leave												
Potential coverage in base scenario^a^ (%)	4.50	*	*	*	*	*	*	*	*	*	*	
Number of women covered	48,664	49,489	50,327	51,178	52,043	52,922	53,815	54,723	55,644	56,581	57,532	582,920
Potential coverage in scenario 1 and 2 (%)	4.50	6.15	7.80	9.45	11.10	12.75	14.40	16.05	17.70	19.35	21	
Number of women covered	48,664	67,635	87,233	107,475	128,374	149,947	172,209	195,178	218,868	243,298	268,484	1,687,364
Estimated number of large and medium companies with lactation room												
No. of additional companies with lactation room	2575	1704	1704	1704	1704	1704	1704	1704	1704	1704	1704	19,623
Percentage out companies with lactation room out of 24,529 total (%)	10.50	17.45	24.40	31.35	38.30	45.25	52.20	59.15	66.10	73.05	80.00	

This table shows the cost of estimation of expanded paid maternity leave

*no change

^a^increase only due to population growth and female labor participation rate

We varied two main parameters to conduct sensitivity analysis for all scenarios (Additional files 2 and 3), namely minimum wage of female employees in formal sectors, and rate of coverage of women eligible to receive paid maternal leave. The details of these scenarios are provided in Table 1.

### The financing need for establishing lactation rooms

The cost of setting up a workplace lactation room was estimated using the standard protocol developed by Alive & Thrive, an initiative managed by FHI 360 and funded by the Bill & Melinda Gates Foundation and other donors [36]. These costs were broken down into the following categories based on the availability of facilities and equipment; US$800 for the basic, US$1000 for the advanced, and US$1200 for the comprehensive workplace lactation room (details of required facilities and equipment are provided in the Alive & Thrive workplace lactation support program toolkit [36]). These figures were multiplied by the number of mid to large, formal sector companies in Indonesia (24,529) using the National Labor Survey data 2012 [27]. The coverage of companies that established a lactation room was gradually increased from 10.5% (current) to 80% from 2020 to 2030 (Table 1). We did not adjust the total costs for inflation over the period. i.e. we conducted the analysis in constant unit cost as of 2014 [36].

### The challenges of implementing maternity protection

To support the quantitative analysis, qualitative interviews were conducted using open-ended questionnaires with stakeholders in five different provinces in Indonesia, namely Serdang Bedagai District (North Sumatra province), Tomohon City (North Sulawesi province), Gianyar District (Bali province), Kupang District (East Nusa Tenggara province), Banda Aceh City and Aceh Jaya District (Aceh province). Stakeholders were chosen using purposive sampling starting from the local district/city health office and follow up was accomplished with snowball sampling where relevant. In total, 29 stakeholders were interviewed between May and October 2016. We interviewed nine staff from District or City Health Offices, nine from district labor and trade offices and labor unions, five from Women and Family Planning Empowerment Boards or offices, four from private sector companies or the chambers of commerce, and two from non-government organizations. The purpose of the interviews was to identify current and potential challenges with improving the existing maternity protection policies by focusing on 6 months paid maternity leave, providing workplace lactation rooms, having a stronger code against BMS marketing, and helping improve local breastfeeding practices (e.g. exclusive breastfeeding period and views on BMS marketing). In this study, stakeholders interviewed ranged from government officials to company and Non-Governmental Organization (NGO) staff. Prior to the interview, respondents were given information regarding the study and were asked to give informed written consent. Interviewers did not transcribe the interview results and directly made real-time summaries of the results. The summaries were then grouped into five themes based on the aim of the interviews (i.e. lactation room, local custom/situation, extending paid leave, legislation, BMS marketing) and further grouped into three main challenges.

## Results

Table 2 presents the estimated number of women eligible for paid maternity leave in the base case and scenario 1 and 2, as well as the estimated number of medium and large companies with lactation rooms. Using the base scenario, the number of women who receive paid maternal leave reaches 57,532 in 2030 a result of population growth and a small increase in the female labor force participation rate. In scenarios 1 and 2, the number of women who receive paid maternal leave in 2030 is 268,484 women, reaching around 1.7 million women in total from 2020 to 2030, allowing for an increase in the proportion of women actually receiving the paid leave to which they are entitled. The number of medium and large firms with lactation rooms is estimated to be 19,623 firms by the end of 2030, reaching 80% of the total number of firms.

### The financing need for paid maternity leave and collectively financed maternity benefits

**Fig. 1:**
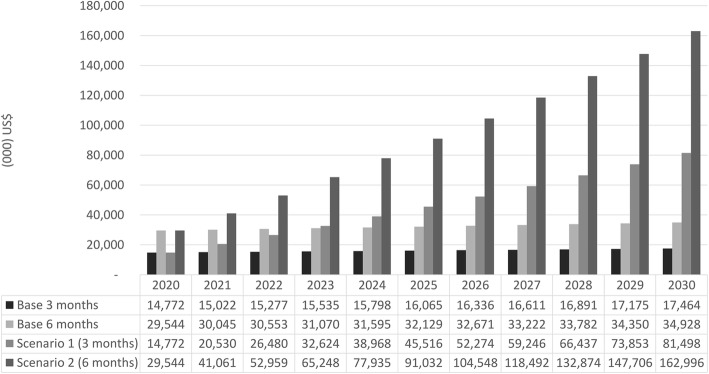
Necessary budget for optimizing paid maternity leave. This figure presents the necessary budget for optimizing paid maternity leave using all scenarios

In the base scenario, which assumed no change in the coverage, the cost of paid maternity leave for the period of 2020 to 2030 was estimated to be US$176.9 million (US$16.1 million/year or US$104.9/mother per year) for 3 months leave and US$353.9 million (US$32.2/year or US$209.7/mother per year) for 6 months leave. In scenario 1 and 2, the total cost of providing three and 6 months of paid maternity leave for 21% of new mothers working in formal sectors in Indonesia from 2020 to 2030 (compared to current coverage of only 4.5%) would increase to US$512.2 million (US$46.6 million/year or US$303.6/mother per year) and US$1.0 billion (US$93.1 million/year or US$607.1/mother per year), as seen in Fig. 1. Using higher coverage estimate of 32% as shown in our sensitivity analysis (Additional file 3), the amount would reach US$735.7 million (US$66.9 million/year or US$436.0/mother per year) for scenario 1 and US$1.5 billion (US$133.8 million/year or US$872.0/mother per year) for scenario 2. If the government were to absorb two-thirds of the cost for 21% coverage, it would cost around US$220.7 million (US$20.1 million/year or US$11.9/mother per year) and US$441.4 million (US$40.1 million/year or US$23.8/mother per year) for scenario 1 and 2, respectively.

### The financing need for creating lactation rooms

An estimated budget is presented in Table 3 to increase access to workplace lactation rooms to the targeted 80% (19,623) of mid to large companies in Indonesia by the end of 2030. The total cost of setting up workplace lactation rooms amounts to US$15.7 million, US$19.6 million and US$23.5 million for the basic, advanced and comprehensive lactation rooms, respectively. The budget is assumed to be covered by the respective companies.

**Table 3:** Cost of setting up lactation room in Indonesia (US$ 000)

Type	2020	2021	2022	2023	2024	2025	2026	2027	2028	2029	2030	Total
Basic	2060.4	1363.8	1363.8	1363.8	1363.8	1363.8	1363.8	1363.8	1363.8	1363.8	1363.8	15,698.6
Advanced	2575.5	1704.8	1704.8	1704.8	1704.8	1704.8	1704.8	1704.8	1704.8	1704.8	1704.8	19,623.2
Comprehensive	3090.7	2045.7	2045.7	2045.7	2045.7	2045.7	2045.7	2045.7	2045.7	2045.7	2045.7	23,547.8

This table shows the estimated cost of setting up lactation room in Indonesia

### The challenges of applying maternity protection policies

The results of the qualitative interviews highlighted that in all regions, both city and district health office staff were aware of the importance of breastfeeding. Participants identified three main inhibiting factors preventing recommended breastfeeding practices. First, BMS have a strong influence on both healthcare workers and society. Some respondents even noted that BMS marketing is so strongly accepted that mothers switch to BMS instead of breastfeeding their children for perceived health benefits. Second, local customs often recommend mothers to introduce complementary food early, rather than practicing recommended exclusive breastfeeding for 6 months, which may hamper efforts to provide breastfeeding according to recommendations. Third, workplace lactation rooms are not commonly available, predominantly due to budgetary constraints. In regard to increasing maternity leave to 6 months, respondents provided mixed responses. Around 24% of our respondents fully agreed, while others worried about the technicalities to implement such policy in their workplace, some were concerned about the potential abandoned tasks in the workplace during leave as well as a potential loss of some form of cash benefit, and some expressed concern that it would reduce their profit. Additional file 4 presents a summary of common regional themes from the face to face interviews.

## Discussion

The combined total costs of providing a full 6 months paid maternity leave, increasing coverage (i.e. enforcing existing legislation) for women, and providing basic lactation rooms in 80% of mid to large firms in Indonesia from 2020 to 2030 would result in a cost of approximately US$1.0 billion (US$616.4/mother per year. This is about 0.1% of the projected nominal Gross National Income (GNI) in 2030 based on Indonesia’s nominal 2016 GNI [37]. The estimated increase in population also contributes to the high cost. However, the projected expenditures in this study are lower than the estimated cost incurred by society (US$1.5 billion) due to cost associated with breastfeeding not according to recommendations, such as treatment of higher rates of childhood pneumonia and diarrhea, and cognitive losses [9]. Furthermore, the figure of US$1.5 billion does not include the value lost through deaths due to breastfeeding not according to recommendations which is estimated at 5377 children (< 2), or the 2000 estimated deaths of women that would be averted by providing breastfeeding according to recommendations [9]. The total potential productivity loss due to these deaths would amount to US$202 million from infant mortality and US$6.7 million from maternal deaths [38], although the number of preventable deaths is the priority concern.

Financing for additional paid maternity leave would help to ensure that mothers had the opportunity for longer maternity leave, and potentially encourage and support more mothers to breastfeed according to recommendations. Indeed, a study has shown that introducing publicly funded paid maternity leave at the minimum wage level may lead to higher breastfeeding rates [39]. This, in turn, may help to avert some of the associated premature child mortality and cognitive losses [5, 40, 41]. A potential impact of an insufficient length of paid maternity leave is that it limits the possibility of providing breastfeeding according to recommendation [3, 5, 42]. If the paid maternity leave policy is implemented optimally and increased to 6 months of leave, the number of deaths averted may be large. We estimated that, if starting from 2020 all new mothers received paid maternity leave for 6 months, the infant mortality rate could be reduced to 16 deaths per 1000 live births from 23 deaths per 1000 live births in 2014 [43]. Thus averting approximately 43,000 infant deaths over the 10 year period (assuming 13% reduction in infant deaths per additional month of paid maternity leave in low and middle income countries, based on the result from Nandi et al. [5]). Thus, the large cost of providing paid maternity leave seems justified and necessary [29]. Furthermore, in the end, policymakers may choose to optimize the current 3 months paid leave policy instead, ensuring that all entitled women are well protected as per regulation, before extending the length of paid maternity leave to 6 months. It is important to note, however, that extending maternity leave may have a positive impact on female labor force participation (up to 140 days) [44]. Further study should explore this aspect, specifically for the case of Indonesia.

This crude estimate of the funds needed by the government if they were to absorb two-thirds of the cost for maternity leave does not capture the budget regularly spent by the government to cover the maternity cash benefit of their employees, which will certainly increase our estimate. As the required budget is potentially large (especially for scenario 2), a further study should be conducted to determine the optimal percentage to be paid by the government, possibly considering the availability of budgets, the share between national and local governments, other government programs, and potential other sources of funding. Furthermore, an important consideration should also be given to the condition where employers could potentially discriminate against female employees if they are to fund all or a large percentage of the maternity leave costs [45], especially within the context of scenario 2. Indonesia should consider collective financing for maternity benefits as it is potentially a more effective approach in securing women’s income during the maternity leave period especially in regards to the detrimental practices against women due to individual employer liability scheme [30]. However, even though the policy governing maternity leave is existing, its implementation remains a challenge [23, 46]. Thus, strong policy enforcement mechanisms need to be in place to ensure that women of childbearing age are able to access maternity leave and do not face discrimination due to the policy implementation [45, 47].

Our interviews yielded some important considerations. Budget constraints are a common barrier to the success of maternity protection programs and the provision of lactation rooms. In addition, lactation rooms do not appear to have strong stakeholder support. A lack of lactation rooms is a persistent issue at the national level [21, 22]. This is critical since some studies have shown that a dedicated breastfeeding space may positively correlate with an increase in exclusive breastfeeding [47–49]. However, more flexible working hours, rather than space at the workplace were preferred in Kupang district, indicating that some areas of the country might have a similar preference and lactation room might not be the best option. This view is shared with a company that we interviewed in Kupang district. Other studies show that lactation rooms may not necessarily be the only solution to ensure the success of providing exclusive breastfeeding. Employers’ and work environment attitude towards breastfeeding and other policies such as flexible working hours and breastfeeding breaks are also important to consider and address [50–54]. Indeed, our respondents stated that mothers may also stop breastfeeding upon returning to work if the encouragement and support for maintaining breastfeeding are lacking. Thus, it is important for the employers to understand the importance of exclusive breastfeeding, for maintaining the health of children which may reduce female employees’ absenteeism from work to care for a sick child [51].

Breast milk substitute marketing also influences the success of maternity protection programs as it encourages nursing mothers to switch from breastmilk to infant formula, or discourages mothers from breastfeeding at all. Also, the stakeholders interviewed were not unanimous in their support for increasing maternity leave to 6 months, which would be critical to pave the way toward increasing maternity protection [29, 55–57]. The experience from Aceh province, the first region in Indonesia to enact the local law of extending maternity leave to 6 months [18], shows other potential challenges and opportunities. While the application of the law is still in its infancy, our interviews confirmed that none of the cities and districts in Aceh province have applied the law, but that some are preparing to do so. Cities and districts here have the autonomy to enact their own local laws, and the governor’s law may or may not necessarily be implemented quickly. Finally, the need for maternity protection and the importance of exclusive breastfeeding must be promoted widely as this is not common practice, and may go against the local social norms and behaviors. Careful coordination between stakeholders (e.g. government offices, firms and labor organizations, NGOs) is also needed to ensure successful implementation of a maternity protection program.

This study has several limitations. First, the assumptions we used for our calculations, e.g., that the programs would start in 2020 and be phased in for 10 years may not align with what might be implemented. The implementation timeframe has significant budgetary implications, once a decision is made and programs are put in place. Nonetheless, the intention of these estimates is to provide a general sense of the cost of paid maternity leave, and how these costs might be best shared between the employers and government. However, we did not simulate other types of maternity benefit financing schemes such as universal coverage, social assistance benefits, and social insurance [58]. In addition, we did not analyze the financial need to expand or implement maternity protection within the informal sector. This may also result in a large financial cost (if not larger than in the formal sector) as more than half of women who work, do so in the informal sector [27]. Indeed, to partially address this need in the informal sector, the Word Breastfeeding Costing Initiative estimated that to provide a maternity entitlement cash benefit to all new mothers living under the poverty line in Indonesia would cost approximately US $296 million per year [59]. This, however, may require further study and the policy recommendation may differ from our current suggestions.

Second, our interviews were only conducted in five different areas. Considering that Indonesia is a vast nation with varied local systems and cultures, the results may not represent Indonesia as a whole. Also, we did not interview mothers who breastfeed as part of our interviews, so we only gained the issues that mothers faced related to breastfeeding from the other stakeholders, mostly from the staff of government offices. However, our results present an overview of the types of challenges likely to be encountered in implementing maternity protection program and provide reasonable estimates for those areas studied, and similar areas across the country.

Third, we did not estimate the possibilities of the share of funding between national and local governments. Further study should explore these possibilities further as it may potentially become a solution to cover the financial need for increased maternity protection (i.e. estimated at US$1.0 billion).

Lastly, we did not discuss other non-economic, ancillary benefits of providing 6 months paid maternity leave that might accrue (e.g. increase uptake of vaccination, increase female labor force participation decrease fertility, and decrease infant mortality rate [40, 44, 60]), rather we strictly focused on associations with providing breastfeeding according to recommendations. Note that we also did not model a direct causal pathway between providing 6 months paid maternity leave and the uptake of breastfeeding, and the subsequent decrease in the cost of not breastfeeding. Rather, we assume there will be benefits from extending maternity protection to the uptake of breastfeeding based on the available literature.

## Conclusions

Although the cost of maternity protection is significant, it is outweighed by the estimated beneficial impact on breastfeeding according to recommendations. The potential decreases in infant and maternal mortality, as well as the economic benefit and cognitive gains in children as a result of breastfeeding per recommendations, are substantial. Mothers require time to properly breastfeed their infants and sharing the cost of paid maternity leave between the private and public sectors may provide a feasible solution to bring maternity protections to new mothers across Indonesia. Various forms of covering the cost of paid maternity leave should be further discussed to find the best options for Indonesia. Challenges in implementing successful maternity protection programs need to be addressed and effective coordination facilitated among government offices, employers, labor organizations and NGOs.

## Additional files

Additional file 1: Calculation of number of women receiving paid maternity leave in 2020. This table shows the calculation of estimating the number of women receiving paid maternity leave in 2020. (DOCX 14 kb)

Additional file 2: Sensitivity analysis result, varying minimum wage. This figure shows the sensitivity analysis result by varying minimum wage. (TIF 2465 kb)

Additional file 3: Sensitivity analysis result, varying rate of coverage of women eligible for receiving paid maternity leave. This figure shows the sensitivity analysis result by varying rate of coverage of women eligible for receiving paid maternity leave. (TIF 2609 kb)

Additional file 4: Interview key points result. This table shows the key points result of the interviews conducted to stakeholders in the regions studied. (DOCX 23 kb)

## Data Availability

The 2010 national census datasets analyzed during the current study are available in the Statistics Indonesia (BPS) repositories, respectively, at https://sp2010.bps.go.id/ All calculation data generated or analyzed during the current study are available from the corresponding author on reasonable request.

## Abbreviations

BMS: Breastmilk substitute
GNI: Gross National Income
ILO: International Labor Organization
LMICs: Low- and middle-income countries
NGO: Non-Governmental Organization
UNICEF: United Nations Children’s Fund
WDI: World Development Indicators
WHO: World Health Organization
