# Supplementary material for: The financing need for expanded maternity protection in Indonesia
Source: Int Breastfeed J. 2019 Jun 25;14:27. doi: 10.1186/s13006-019-0221-1 (PMC6593591; doi:10.1186/s13006-019-0221-1)
Supplement: Supplementary file 1 — Calculation of number of women receiving paid maternity leave in 2020. This table shows the calculation of estimating the number of women receiving paid maternity leave in 2020. (DOCX 14 kb) [file 13006_2019_221_MOESM1_ESM.docx]

**Additional file A. Calculation of number of women receiving paid maternity leave in 2020**

| Total population of children between 0 - 11 months age in 2010* [32] | Yearly population growth of Indonesia [31] | Total population of children between 0 - 11 months age in 2020 | Female labor participation rate (2017) [6] | Increase of female labor participation rate from 2016 to 2017 | Estimated female labor participation rate in 2020 | Women working in formal sector in 2020** [26] | Coverage of female receiving paid maternity leave in 2020*** [1] | Number of women receiving paid maternity leave in 2020 |
| --- | --- | --- | --- | --- | --- | --- | --- | --- |
|  |  | a |  |  | b | c | d | a*b*c*d |
| 4,398,405 | 1.30% | 5,004,834 | 50.70% | 0.20% | 51.30% | 42.12% | 4.50% | 48,664 |

*As a proxy for women who should be exclusively breastfeeding their children

**Assumed to be constant since 2012

***Assumed to be constant since 2010

This table shows the calculation of estimating the number of women receiving paid maternity leave in 2020
