# Supplementary figures and images for: The financing need for expanded maternity protection in Indonesia
Source: Int Breastfeed J. 2019 Jun 25;14:27. doi: 10.1186/s13006-019-0221-1 (PMC6593591; doi:10.1186/s13006-019-0221-1)

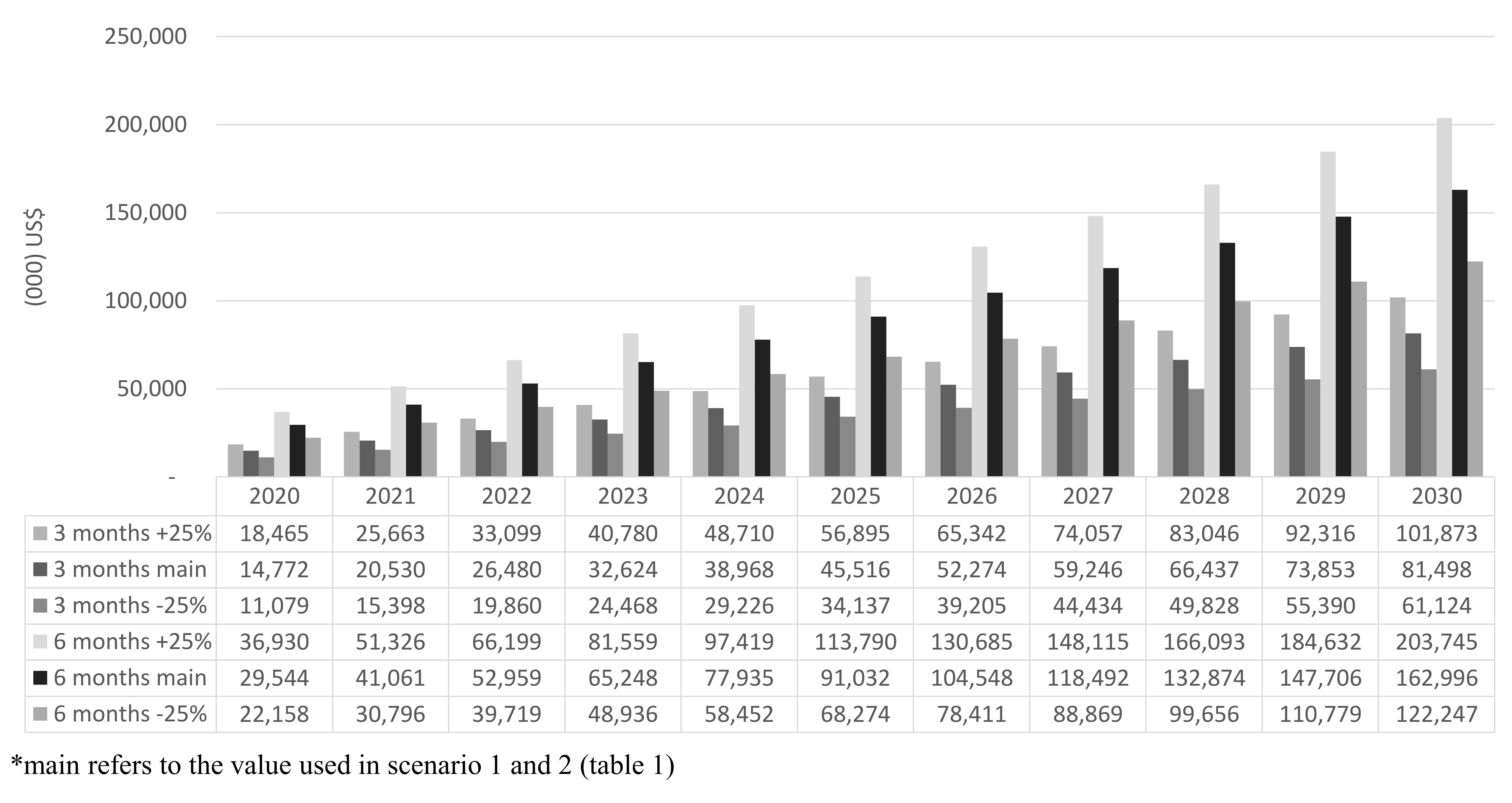

Supplement: Supplementary file 2 — Sensitivity analysis result, varying minimum wage. This figure shows the sensitivity analysis result by varying minimum wage. (TIF 2465 kb) [file 13006_2019_221_MOESM2_ESM.tif]

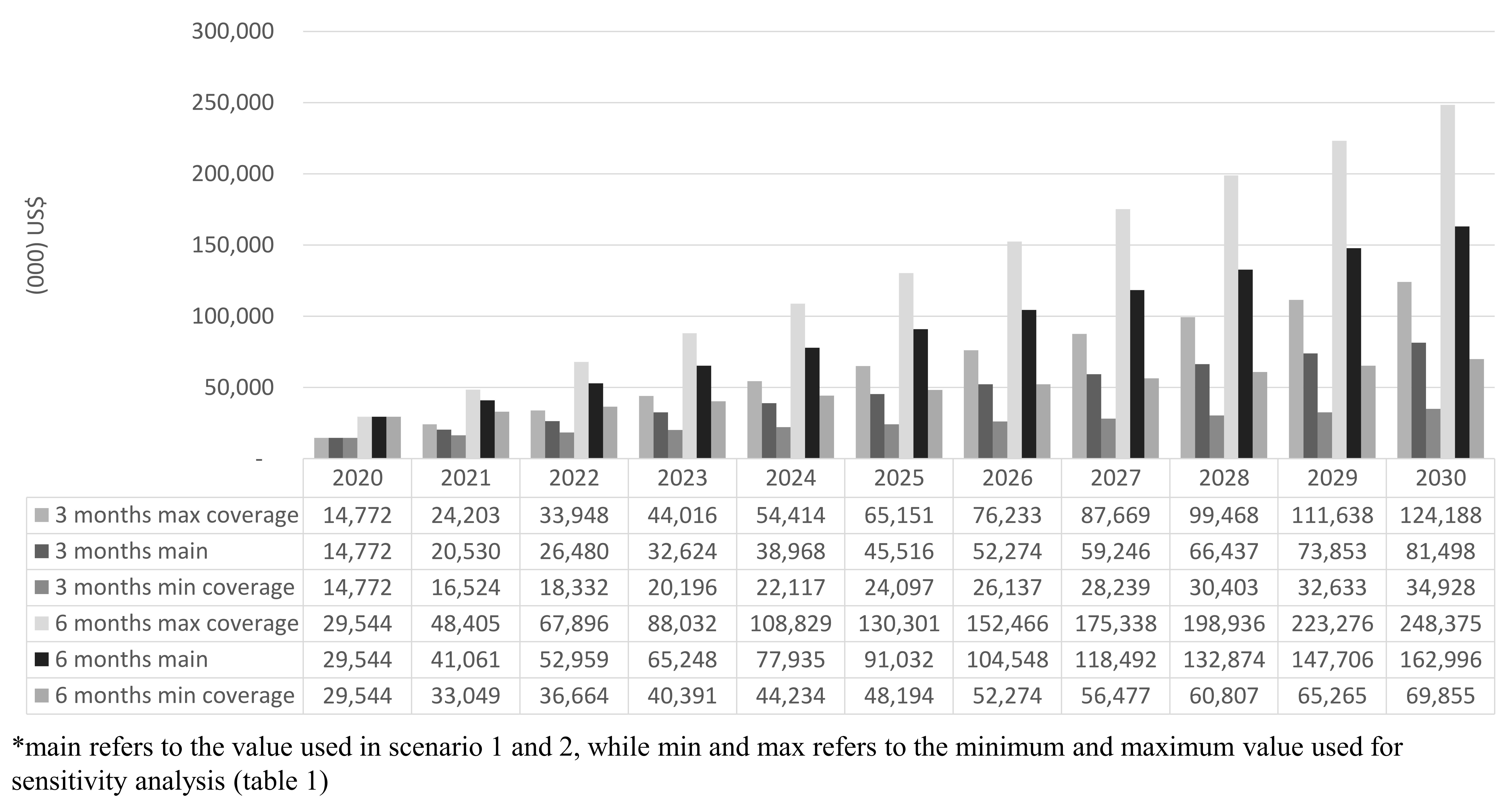

Supplement: Supplementary file 3 — Sensitivity analysis result, varying rate of coverage of women eligible for receiving paid maternity leave. This figure shows the sensitivity analysis result by varying rate of coverage of women eligible for receiving paid maternity leave. (TIF 2609 kb) [file 13006_2019_221_MOESM3_ESM.tif]
