# Supplementary material for: The financing need for expanded maternity protection in Indonesia
Source: Int Breastfeed J. 2019 Jun 25;14:27. doi: 10.1186/s13006-019-0221-1 (PMC6593591; doi:10.1186/s13006-019-0221-1)
Supplement: Supplementary file 4 — Interview key points result. This table shows the key points result of the interviews conducted to stakeholders in the regions studied. (DOCX 23 kb) [file 13006_2019_221_MOESM4_ESM.docx]

| **Additional file D. Interview key points result** | | | | | | | | | | |
| --- | --- | --- | --- | --- | --- | --- | --- | --- | --- | --- |
| City/district |  | Key point | | | | | | | | |
|  |  | Lactation room |  | Local custom/situation |  | Extending paid leave |  | Legislation |  | BMS marketing |
| Serdang Bedagai |  | - Budget constraints limit the possibility of providing lactation rooms |  | - People’s knowledge about breastfeeding is incomplete - People who can afford formula milk are viewed as having higher social status |  | - Local district health office basically agrees with increasing the length of paid maternity leave, but they are unsure about the technicalities and the potential unfinished office work during the leave |  | - District health office has issued administrative punishment for midwives who sold formula milk to nursing mothers |  | - Formula milk companies provide rewards to health staff who successfully sell formula milk to (nursing) mothers - BMS marketing has cause midwives to avoid suggesting mothers to provide early breastfeeding initiation |
| Tomohon |  | - Does not yet have local regulations on lactation room |  | - The secretary of the district health office in Tomohon have been fully informed about maternity protection, working women’s rights, and exclusive breastfeeding. - The general public is not fully aware of the importance of exclusive breastfeeding so there is no movement to support it yet |  | - Maternity leave for mothers giving birth has been implemented and the salaries of employees on maternity leave are paid accordingly in public sector. In the private sector, however, it is currently only applied in larger companies - The maternity protection program is constrained by budget issue - Tri-party relationship between the government, companies and unions exists, and they are ready to support maternity protection programs |  | - Currently, there are no local laws or regulations in support of breastfeeding - Policymakers do not yet provide support for breastfeeding legislation - Until now there has been no specific local regulation on the sale and promotion of infant formula |  | - The strong influence of formula milk companies and their aggressive advertisements may cause women to choose to stop breastfeeding and use formula milk |
| Gianyar |  | - No local government regulation on lactation rooms - Budget constraint limit the possibility of providing lactation rooms |  | - The local chamber of commerce members personally supports giving up to 4 months of paid maternity leave, but this has not yet been practiced - The preference for feeding an infant formula milk is similar across income group - Main reasons why women choose to use formula milk are due to returning to work or maintaining the shape of their bodies |  | - The concept of 6 months paid maternity leave received positive views from institutions |  | - Although government officials have been fully informed about the maternity protection, working women’s rights, and exclusive breastfeeding, turning policy into law is a challenge |  |  |

| **Additional file D. Interview key points result (continued)** | | | | | | | | | | |
| --- | --- | --- | --- | --- | --- | --- | --- | --- | --- | --- |
| City/district |  | Key point | | | | | | | | |
|  |  | Lactation room |  | Local custom/situation |  | Extending paid leave |  | Legislation |  | BMS marketing |
| Kupang |  | - The provision of lactation rooms in formal sectors are not viewed as necessary as mothers have flexible working time even after their maternity leave period (usually three months after delivery) is over, e.g. they can leave work two hours earlier - A company, however, is in the process of setting up a lactation room |  | - In one company, maternity cash benefits are paid higher than local minimum wage. Also, the company allows longer lunch breaks for women who breastfed so they can go home and breastfed their infant. The company is currently building a new site which they said will have lactation rooms - It is commonly believed that formula milk is better than breastmilk - The older generation still believe that food (e.g. banana and papaya) are good for 3 month old babies, and mothers usually comply - Primary health centers (*puskesmas*) are unable to provide refrigerator specifically to store breastmilk for their employees due to lacking capacity of electricity power, and they do not have the funding to raise their electricity capacity. - Most women work in the informal sector, therefore they are not protected by maternity leave policy. - Lack of exclusive breastfeeding counselling due to the issues with infrastructure and land contours, e.g. some areas cannot be reached because they are still surrounded by mountains and they have no proper infrastructure such as road, water, electricity, and have no nearby primary health center. - District health office provides advertisement in radio about the importance of breastfeeding twice a day |  | - Increasing paid maternity leave to 6 months is supported by officials, but has a skeptical response from factories as it will affect their profit. They view that flexible working time is sufficient. |  | - A local law requiring mothers to be attended by health care staff during delivery to ensure early breastfeeding initiation has been prepared by district health office and already signed by the Regent |  | - BMS marketing is not allowed by the district health office in primary health centers and hospitals |

| **Additional file D. Interview key points result (continued)** | | | | | | | | | | |
| --- | --- | --- | --- | --- | --- | --- | --- | --- | --- | --- |
| City/district |  | Key point | | | | | | | | |
|  |  | Lactation room |  | Local custom/situation |  | Extending paid leave |  | Legislation |  | BMS marketing |
| Banda Aceh City and Aceh Jaya District |  | - Lactation rooms are rarely provided in offices and companies. |  | - Through the implementation of Islamic law, it is suggested (*sunnah*) that infants should be breastfed up to two years. - Higher income women may choose to give formula milk to infants because they are constrained by work hours (does not have time to breastfeed their children of express breastmilk) and believe that formula milk can replace the nutrition from breastmilk. - The society in Aceh already knows the benefits of providing exclusive breastfeeding. It is good for the health and intelligence for babies, and good for emotional bonding between babies and mothers. |  | - The Governor Law of extending maternity leave to six months receives mixed views. While some government offices support the law, some others have concerns regarding it, e.g low number of workers in the office, so works will be abandoned during maternity leave; three months leave and flexible working time afterwards is deemed sufficient; may create burden or loss to companies. - Local legislative body and labor union fully support the new Governor Law. |  | - Governor Law in Aceh Province on maternity protection has been initiated since 2010 as *Sharia* law, and enacted as governor regulation in 2016 and require companies and offices to provide 6 months maternity leave. However, no cities/district in the province have applied the Governor Law yet, but some are in the preparation to do so. - Local laws known as *Qonun* encourage people to provide exclusive breastfeeding. - Local government regulation banning health care staff to recommend nursing mothers to consume formula milk is available. - Local government regulation banning BMS advertisement is available. |  | - There is no regulation on the prohibition of BMS sales in Aceh Province, yet. - Formula milk companies usually participate in family gathering and give gifts. |
